# Ershiwuwei Shanhu Pill ameliorates cognitive impairment in Alzheimer’s disease mice by remodeling gut microbiota and host serum metabolites

**DOI:** 10.3389/fnagi.2026.1820691

**Published:** 2026-06-19

**Authors:** Ning Li, Kunxia Jiang, Xiaoyuan Peng, Ce Yang, Wei Xiong, Wenxiang Wang

**Affiliations:** 1 Chongqing Three Gorges Medical College, Chongqing, China; 2 Chongqing Key Laboratory of Development and Utilization of Genuine Medicinal Materials in Three Gorges Reservoir Area, Chongqing, China

**Keywords:** 16S rRNA sequencing, Alzheimer’s disease, Ershiwuwei Shanhu Pill, gut microbiota-metabolite-brain axis, metabolomics analysis

## Abstract

Alzheimer’s disease (AD), with the most prominent pathological feature of the accumulation of amyloid-beta (Aβ) plaques and neurofibrillary tangles of hyperphosphorylated tau proteins (P-tau), is the foremost cause of dementia. Ershiwuwei Shanhu Pill (ESP) is commonly used in clinical practice in Tibetan areas to treat AD, and has been shown to alleviate cognitive impairment. However, whether ESP exerts its therapeutic effects by modulating the gut microbiota and serum metabolites remains unclear. In this study, APPswe/PSEN1dE9 transgenic (APP/PS1) mice (*n* = 11 per group) were treated with ESP (200 mg/kg, oral gavage) daily for 2 months and evaluated using the Morris Water Maze (MWM), brain histopathology, immunofluorescence, 16S rRNA sequencing, and untargeted serum metabolomics. ESP improved cognitive performance, reduced Aβ deposition and P-tau levels, and attenuated neuroinflammation. Concurrently, ESP significantly reshaped the gut microbiota (e.g., increasing *Dubosiella* and decreasing *Bacteroides*) and altered serum metabolites involved in tryptophan metabolism and glycolysis pathways (e.g., elevating Fructose 1,6-bisphosphate and reducing N-Acetylserotonin). In conclusion, these neuroprotective effects of ESP are associated with a remodeling of the gut microbiota and metabolic profile, providing a pharmacological basis for its clinical application and novel insights into AD intervention via the gut microbiota-metabolite-brain axis.

## 1 Introduction

Alzheimer’s disease (AD), a progressive neurodegenerative disorder and the foremost cause of dementia, is characterized by irreversible cognitive decline, behavioral disturbances, and significant neuronal loss, and represents a significant global health challenge (Qi et al., 2025). Although the initial pathogenic mechanisms remain incompletely understood, AD is primarily driven by the accumulation of amyloid-beta (Aβ) plaques, neurofibrillary tangles composed of hyperphosphorylated tau proteins (P-tau), and neuroinflammation (Kimura et al., 2025; Topol, 2025). Neuropathological abnormalities in AD may begin 15–30 years before the onset of clinical manifestations (Jansen et al., 2015; Jia et al., 2024).

Microglia have been suggested as key participants in AD (Heneka et al., 2025). As the resident immune cells of the central nervous system, microglia play crucial roles in maintaining homeostasis, responding to injury, and modulating inflammation (Chen Y. et al., 2025). Extracellular Aβ and/or intraneuronal P-tau in AD can activate microglia (Merighi et al., 2022). In the early stages of AD, microglia become activated and cluster around Aβ plaques, involving in eliminating Aβ through phagocytosis and containment of pathological spread, thus exerting a neuroprotective effect (He et al., 2025; Sun et al., 2024). However, sustained activation in AD transforms microglia into a chronic pro-inflammatory state, resulting in the phagocytosis impairment and the release of cytotoxic cytokines, which exacerbates a vicious cycle of synapse loss, tau pathology and neuronal dysfunction (AmeliMojarad and AmeliMojarad, 2024; Heneka et al., 2015).

In addition to the three characteristic pathological features mentioned above, the etiology of AD also includes gene mutations (Krishnamurthy et al., 2025), synaptic damage (Chaubey et al., 2025), mitochondrial dysfunction (Yang et al., 2025), metabolic abnormalities (Song et al., 2025), and other age-related changes (Butovsky and Rosenzweig, 2025). Given the complexity of AD pathology, therapeutic approaches focusing on a single target, such as Aβ or P-tau, have consistently shown high failure rates in clinical trials, highlighting the urgent need for multi-target strategies, combination therapies, or systems-based interventions that address the interconnected pathways of neurodegeneration.

Traditional Chinese medicine, particularly herbal formulations with a history of use in cognitive disorders, represents a rich source of multi-target therapeutic approaches. Within this broad tradition, Tibetan medicine, with its unique theoretical framework, offers candidates such as Ershiwuwei Shanhu Pill (ESP). ESP is a traditional classic prescription developed in the 18th century and is recorded in the *Pharmacopoeia of the People’s Republic of China* (2020 Edition, Volume 1) (Chinese Pharmacopoeia Commission, 2020). ESP, with the effect of inducing resuscitation and dredging collaterals, has been mainly used to treat White Channel Disease (དཀར་རྩ་ནད, dkar rtsa nad, a neuropathic disorder in Tibetan medicine), including AD. Phytochemical investigations have revealed that ESP contains a wide array of compounds, including flavonoids and their glycosides (e.g., liquiritin, isoliquiritigenin, kaempferitrin, liquiritigenin, vitexin, kaempferol, rutin, isoglycyrrhiziofuranoside, neoliquiritin, hydroxysafflor yellow A, wogonoside, apigenin, apigenin glucuronide, isoliquiritin, isorhamnetin, acacetin, isokaempferide, formononetin, licoflavone A, licoricone, and luteolin), alkaloids (e.g., berberrubine), terpenoids and their glycosides (e.g., sweroside, swertiachoside B, amaroswerin, arjunglucoside I, amarogentin, loganic acid, geniposidic acid, arjungenin, terminolic acid, caulophyllogenin, dehydrocostus lactone, 18β-glycyrrhetinic acid, and oleanolic acid), phenolic and organic acids (e.g., gallic acid, shikimic acid, quinic acid, syringic acid, chlorogenic acid, cryptochlorogenic acid, and caffeic acid), as well as fatty acids (e.g., linolenic acid), apocarotenoids (e.g., crocetin), chromones, coumarins, and esters (Han et al., 2025). Among them, chlorogenic acid (Li Z. et al., 2023), luteolin (Daily et al., 2021), crocin (Zou et al., 2025), and quinic acid (Li et al., 2024) have shown neuroprotective effects on AD model mice through modulation of gut microbiota. Our preliminary animal experiments have confirmed that ESP administration can improve cognitive performance in AD mice model, which is consistent with existing literature results (Tsering et al., 2022).

However, existing studies are siloed, and the effects of ESP on the gut microbiota, neuroinflammation, Aβ, or P-tau pathology are in isolation. Whether the modulation of the gut microbiota by ESP leads to alterations in host serum metabolites, which in turn regulate microglial function to ameliorate Aβ and tau pathology. Therefore, this study aimed to investigate the integrated “gut microbiota-serum metabolite-microglia” axis as a central mechanism underlying the neuroprotective effects of ESP in AD. Behavioral tests, 16S rRNA gene sequencing of gut microbiota, untargeted serum metabolomics, pathological staining, and immunofluorescence analyses of Aβ deposition, tau pathology, and microglial activation were performed to confirm the efficacy of ESP in mitigating cognitive decline and neuropathology, identify specific changes in the gut microbial community and associated serum metabolite profiles induced by ESP, and correlate these peripheral changes with the gut microbiota.

## 2 Materials and methods

### 2.1 Experimental Animals and treatment

Six-month-old male SPF-grade APPswe/PSEN1dE9 transgenic (APP/PS1) mice (*n* = 22) and 6-month-old male C57BL/6J wild-type (WT) mice (*n* = 11) were purchased from Jiangsu Huachuang Sinno Pharmaceutical Technology Co., Ltd. (Animal license no. SCXK (Su) 2020-0009). All animals were housed in the SPF animal laboratory of Chongqing Three Gorges Medical College under controlled conditions, with a 12-h light/dark cycle (lights on from 8:00 to 20:00) at 24°C. The mice had unrestricted access to standard chow and water. Before the experiment, all animals underwent a 1-week adaptation period. APP/PS1 mice were randomly assigned to two groups: the model group (APP/PS1) and the ESP group (200 mg/kg). The WT mice served as the normal control group. ESP was obtained from Jinhe Tibetan Medicine Co., Ltd. (National Medicine Standard No. Z63020059, Batch No. 01230616). All animals underwent daily oral gavage for 2 months. At the end of the dosing period, a 5-day Morris Water Maze (MWM) behavioral test was conducted. Following the final test, all mice were euthanized, and fresh stool, serum, and brain tissue samples were collected for further analysis.

### 2.2 MWM test

The MWM test was used to evaluate spatial learning and memory capabilities (Lei et al., 2025; Wei et al., 2025). The mice were placed into a circular tank with a diameter of 1.2 m, filled with opaque water mixed evenly with non-toxic milk powder at a depth of 40 cm, and maintained at a temperature of 23 ± 2°C. The pool was divided equally into four quadrants, and a platform (9 cm diameter) submerged 1 cm below the water surface was fixed in one of the quadrants. Each mouse underwent four trials per day for five consecutive days. Each mouse was positioned at points in different quadrants and was allowed 60 s to find the platform and stay there for 15 s. If the mouse failed to locate the platform within 60 s, it was manually guided to the platform and allowed to stay on it for 15 s. The platform was removed 24 h after the last training trial, and each mouse was placed into the pool and observed for 60 s. All swimming activities were automatically recorded using the ZS001 animal behavior analysis system (Beijing Zhongshi Technology Co., Ltd.).

### 2.3 Congo red staining and thioflavin-S staining

Congo red staining and thioflavin-S staining were used to analyze the accumulation of Aβ (Wang Q. et al., 2025). For Congo red staining, the brain sections were deparaffinized with xylene, rehydrated with graded ethanol solutions, stained with Congo red (G1056, Servicebio) overnight, soaked in hematoxylin dye solution (G1004, Servicebio) for 60 s, and sealed with neutral resin. Finally, images were captured using a VS200 Virtual Slide Microscope (Olympus, Japan). For Thioflavin-S staining, the brain paraffin sections were dewaxed conventionally in water, stained with 0.3% Thioflavin-S solution (Thioflavin S, Sigma, T1892) for 8 min, washed with 80% alcohol, and soaked in DAPI (G1012, Servicebio) for 10 min. Finally, anti-fluorescence quenching sealing tablets (G1401, Servicebio) were used to seal the slide. Images were acquired under a Nikon Eclipse C1 upright fluorescence microscope (Nikon Corporation, Tokyo, Japan).

### 2.4 Immunofluorescence assay

Immunofluorescence assays were performed as described previously (Li et al., 2021). After dewaxing and antigen repair, brain sections were blocked within 3% BSA (GC305006, Servicebio) for 30 min at room temperature. Next, the brain sections were incubated with primary antibodies overnight at 4°C and then incubated with the corresponding secondary antibodies. Primary antibodies included Aβ_1–42_ (1:400, GB115755, Servicebio), P-tau (1:600, GB113883, Servicebio), and Iba1 (1:500, GB15105, Servicebio). Cy3 conjugated Donkey Anti-Rabbit IgG (H + L) (1:300, GB21403, Servicebio) or Alexa Fluor^®^ 488-conjugated Donkey Anti-Goat IgG (H + L) (1:300, GB25404, Servicebio) were used as secondary antibodies. Brain sections were then incubated with DAPI staining solution (G1012, Servicebio), an autofluorescence quencher (G1221, Servicebio), and sealed with an antifluorescent quencher (G1401, Servicebio). Brain sections were washed three times with PBS (G4250, Servicebio) between every two steps. Images were captured using a Nikon Eclipse C1 upright fluorescence microscope. Fluorescence intensity was analyzed using ImageJ software.

### 2.5 Fecal 16S rRNA sequencing

Fecal samples were collected for 16S rRNA sequencing, as previously described (Li et al., 2026). DNA was extracted from fecal samples using the cetyltrimethylammonium bromide (CTAB) method. Subsequently, the concentration and purity of the extracted DNA were determined using 1.2% agarose gel electrophoresis. The hypervariable V3-V4 region of the 16S rRNA gene was amplified with the primer pairs 338F (5′-ACTCCTACGGGAGGCAGCA-3′) and 806R (5′-GGACTACHVGGGTWTCTAAT -3′). The PCR products were purified and quantified. Purified amplicons were sequenced using an Illumina MiSeq platform (Illumina, San Diego, CA, United States). The denoising DADA2 sequences of fecal microbiota, annotating species taxonomy, and leveling ASV/OTU abundance were using QIIME2 (2019.4). Microbial functions were predicted using PICRUSt2. The analysis results were visualized using the R Package (v3.2.0), including species composition bar charts, Alpha diversity, Beta diversity, species composition heatmaps, differential species expression heatmaps, scatter plots, volcano plots, metabolic pathway statistical charts, and other graphs.

### 2.6 Metabolomics

Untargeted metabolomics was performed to identify the differential metabolites in each group of mice (Li et al., 2025). After thoroughly mixing the thawed serum samples with pre-chilled (−20°C) LC-MS grade methanol (1:4 v/v), the resulting mixtures were centrifuged. The obtained supernatants were subjected to vacuum freeze-drying, and the dried residues were dissolved in 150 μL of 80% methanol containing 2-chloro-L-phenylalanine (4 ppm). Finally, the supernatants were filtered separately using 0.22 μm microporous membrane filters for liquid chromatography-mass spectrometry (LC-MS) detection.

Liquid chromatography conditions (Chao et al., 2022; Li H. Y. et al., 2023): LC analysis was performed using a Vanquish UHPLC System (Thermo Fisher Scientific, United States). Chromatography was performed using an ACQUITY UPLC^®^ HSS T3 (2.1 × 100 mm, 1.8 μm) (Waters, Milford, MA, United States). The column was maintained at 40°C. The flow rate and injection volume were set at 0.3 mL/min and 2 μL, respectively. For LC-ESI (+)-MS analysis, the mobile phases consisted of (B2) 0.1% formic acid in acetonitrile (v/v) and (A2) 0.1% formic acid in water (v/v). Separation was conducted under the following gradient: 0∼1 min, 10% B2; 1∼5 min, 10∼98% B2; 5∼6.5 min, 98% B2; 6.5∼6.6 min, 98∼10% B2; 6.6∼8 min, 10% B2. For LC-ESI (-)-MS analysis, the analytes were dissolved in (B3) acetonitrile and (A3) ammonium formate (5 mM). Separation was conducted under the following gradient: 0∼1 min, 10% B3; 1∼5 min, 10∼98% B3; 5∼6.5 min, 98% B3; 6.5∼6.6 min, 98∼10% B3; 6.6∼8 min, 10% B3.

Mass spectrum conditions (Li H. Y. et al., 2023): Mass spectrometric detection of metabolites was performed on an Orbitrap Exploris 120 (Thermo Fisher Scientific, United States) with an ESI ion source. Simultaneous MS1 and MS/MS (Full MS-ddMS2 mode, data-dependent MS/MS) acquisition was used. The parameters were as follows: sheath gas pressure, 40 arb; aux gas flow, 10 arb; spray voltage, 3.50 kV and −2.50 kV for ESI(+) and ESI(−), respectively; capillary temperature, 325°C; MS1 range, m/z 100–1,000; MS1 resolving power, 60,000 FWHM; number of data-dependent scans per cycle, 4; MS/MS resolving power, 15,000 FWHM; normalized collision energy, 30%; dynamic exclusion time, automatic.

The raw data were first converted to mzXML format using the ProteoWizard software package (version 3.0.8789) (Rasmussen et al., 2022), and were processed using R XCMS (v3.12.0) for feature detection, retention time correction, and alignment (Navarro-Reig et al., 2015). Metabolites with RSD > 30% in quality control (QC) samples were filtered. Metabolites (mass < 30 ppm) were identified based on MS/MS data, which were matched with several databases, including the Human Metabolome Database (HMDB)^1^ (Wishart et al., 2022), MassBank^2^ (Neumann et al., 2025), LIPID Metabolites and Pathways Strategy (LIPID MAPS)^3^ (Conroy et al., 2024), mzCloud^4^ (Chernonosov et al., 2024), Kyoto Encyclopedia of Genes and Genomes (KEGG)^5^ (Kanehisa et al., 2023), and the metabolite database self-developed by Panomix Biomedical Tech Co., Ltd. (Shuzhou, China).

Multivariate data analysis, including principal component analysis (PCA), partial least squares discrimination analysis (PLS-DA), and orthogonal partial least squares-discriminant analysis (OPLS-DA), were conducted using the R Ropls (v1.22.0) package (Wang J. et al., 2025). Variable importance for projection (VIP) was calculated for the OPLS-DA first principal component variable importance projection. Significant differential metabolites were screened with *p* < 0.05 and VIP > 1.0. Differential metabolites were subjected to KEGG pathway enrichment analysis using the hypergeometric test (Zhang et al., 2025).

### 2.7 Correlation analysis between microbiota and metabolites

Spearman correlation analysis was performed to analyze the relationships between differential metabolites and differential gut microbiota in the WT, APP/PS1, and ESP groups. Heatmaps were plotted using the BioDeep Platform^6^ and SRplot^7^ (Tang et al., 2023).

### 2.8 Statistical analysis

All data are represented as mean ± standard error of the mean (SEM). Statistical analyses were performed using GraphPad Prism 10.1.2 (GraphPad Software, United States). An unpaired *t*-test was used for comparison between two groups, and one-way analysis of variance (ANOVA) among multiple groups. Statistical significance is represented as *p < 0.05, **p < 0.01, and ***p < 0.001.

## 3 Results

### 3.1 ESP alleviated neuroinflammation and restored impaired cognitive functions of APP/PS1 mice

**FIGURE 1:**
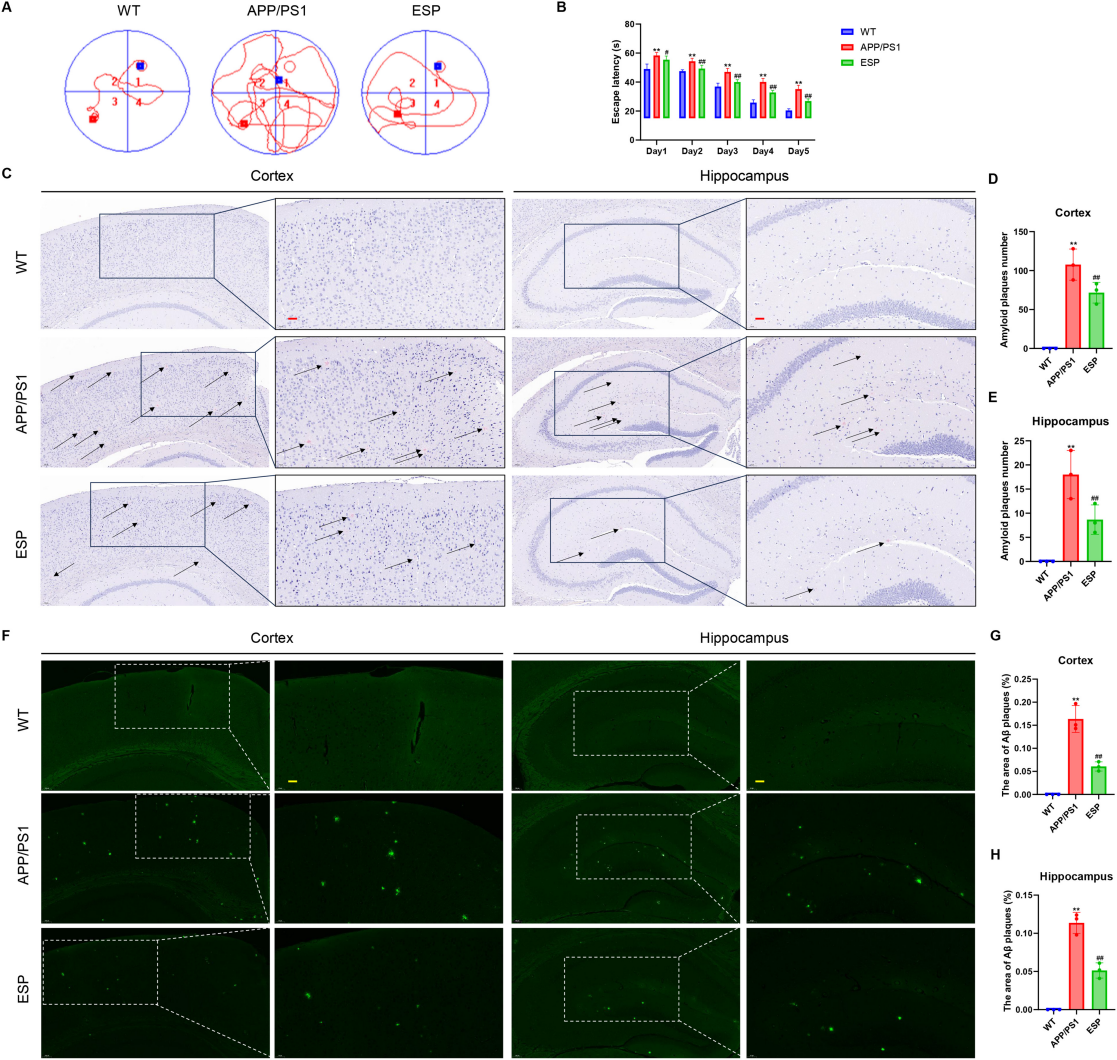
ESP ameliorated cognitive impairment and alleviated Aβ plaques deposition in APP/PS1 mice. **(A)** Representative escape paths in MWM. The starting point is in the third quadrant and the platform is in the first quadrant. **(B)** Escape latency of MWM in 5 days (*n* = 11). **(C)** Representative images of Congo red staining, the stained spots were marked with black arrows. Quantification analysis of Congo red staining in the hippocampus **(D)** and cortex **(E)** (*n* = 3). **(F)** Representative images of thioflavin-S staining. Quantification analysis of thioflavin-S staining in the hippocampus **(G)** and cortex **(H)** (*n* = 3). Data were presented as mean ± SEM. ^##^*p* < 0.01 compared with the WT group. **p < 0.01 compared with the APP/PS1 group. Bar = 50 μm.

MWM was used to evaluate the spatial learning and memory abilities of mice. Compared to the WT mice, the APP/PS1 mice required more time to find the platform, whereas ESP significantly reduced the escape latency (*p* < 0.01) (Figures 1A,B). To assess the potential beneficial inhibitory effect of ESP on Aβ accumulation, Congo red staining and thioflavin-S staining were performed to analyze the number and area of Aβ. Compared to the WT mice, the APP/PS1 mice showed a remarkable increase in Aβ plaques in the hippocampus and cortex, while ESP markedly alleviated Aβ plaques deposition (*p* < 0.01) (Figures 1C–H).

**FIGURE 2:**
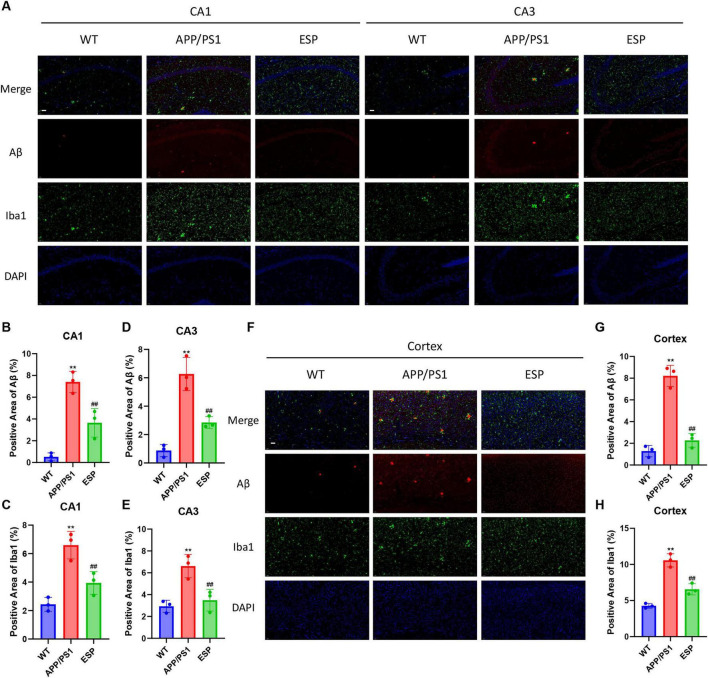
ESP alleviated neuroinflammation and reduced Aβ aggregation in hippocampus and cortex of APP/PS1 mice. Immunofluorescence double-labeling were used to detect the Aβ (red) and Iba1 (green) expression. Representative microscopic images of Aβ and Iba1 in the hippocampus subregions (CA1 and CA3) **(A)** and cortex **(F)**. Quantification of the Aβ **(B)** and Iba1 **(C)** in CA1. Quantification of the Aβ **(D)** and Iba1 **(E)** in CA3. Quantification of the Aβ **(G)** and Iba1 **(H)** in cortex. Data were presented as mean ± SEM. ^##^*p* < 0.01 compared with the WT group. **p < 0.01 compared with the APP/PS1 group. *n* = 3. Bar = 50 μm.

**FIGURE 3:**
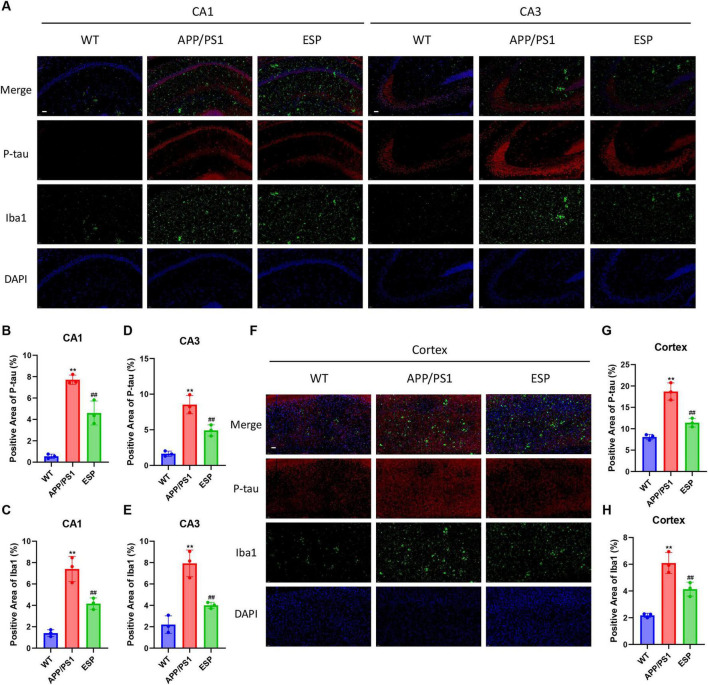
ESP alleviated neuroinflammation and ameliorated tau pathology in hippocampus and cortex of APP/PS1 mice. Immunofluorescence double-labeling were used to detect the P-tau (red) and Iba1 (green) expression. Representative microscopic images of P-tau and Iba1 in the hippocampus subregions (CA1and CA3) **(A)** and cortex (F). Quantification of the P-tau **(B)** and Iba1 **(C)** in CA1. Quantification of the P-tau **(D)** and Iba1 **(E)** in CA3. Quantification of the P-tau **(G)** and Iba1 **(H)** in cortex. Data were presented as mean ± SEM. ^##^*p* < 0.01 compared with the WT group. **p < 0.01 compared with the APP/PS1 group. *n* = 3. Bar = 50 μm.

Microglia-mediated neuroinflammation plays a pivotal role in the pathogenesis of AD. Immunofluorescence was performed to evaluate the activation of microglia, Aβ accumulation, and tau pathology. Compared to the WT mice, the APP/PS1 mice showed a remarkable increase in Iba1, an emblem of microglial activation, while ESP dramatically decreased the Iba1 level, which was positively correlated with Aβ deposition and P-tau (*p* < 0.01) (Figures 2A–H, 3A–H).

### 3.2 ESP regulated the fecal microbiota of APP/PS1 mice

**FIGURE 4:**
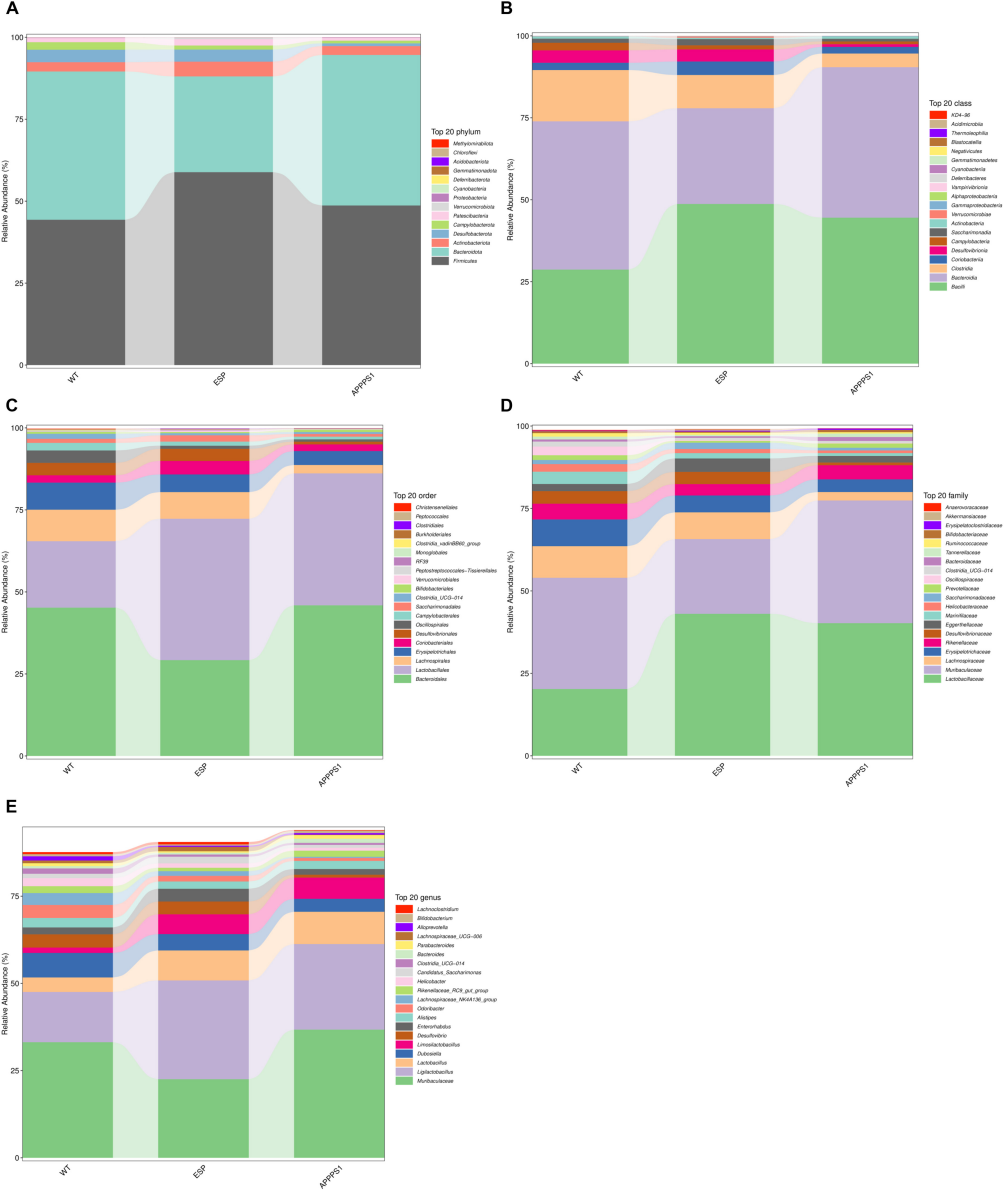
ESP altered fecal microbiota composition in APP/PS1 mice. **(A–E)** Column charts of top 20 species composition of fecal microbiota at the level of phylum (A), class **(B)**, order **(C)**, family **(D)**, and genus **(E)** respectively among the WT, APP/PS1, and ESP groups. The horizontal axis represents different experimental groups, while the vertical axis represents the relative abundance values of species composition at different levels. *n* = 8.

To accurately the restorative effects of ESP treatment on gut microbiota dysbiosis in APP/PS1 mice, we performed 16S rRNA gene sequencing on fecal samples of the WT, APP/PS1, and ESP groups. The composition of the fecal microbiota across groups is summarized in Figures 4A–E, which displays the top 20 most abundant taxa at each taxonomic level (phylum to genus). At the genus level, significant alterations were observed in the APP/PS1 group compared to the WT group. Specifically, the relative abundances of several genera, including *Bifidobacterium, Parabacteroides, Bacteroides, Limosilactobacillus, Lactobacillus*, and *Muribaculaceae* were higher in the APP/PS1 group compared to the WT group. Additionally, the relative abundance of *Lachnoclostridium, Alloprevotella, Lachnospiraceae_UCG−006, Clostridia_UCG−014, Candidatus_Saccharimonas, Helicobacter, Lachnospiraceae_NK4A136_group, Odoribacter, Enterorhabdus, Desulfovibrio*, and *Dubosiella* were lower in the APP/PS1 group compared to the WT group. Notably, ESP treatment largely reversed these alterations. Furthermore, the relative abundance of *Rikenellaceae_RC9_gut_group* and *Alistipes* were reduced, and the relative abundance of *Ligilactobacillus* was elevated in the APP/PS1 group compared to the WT, while ESP exacerbated this trend.

**FIGURE 5:**
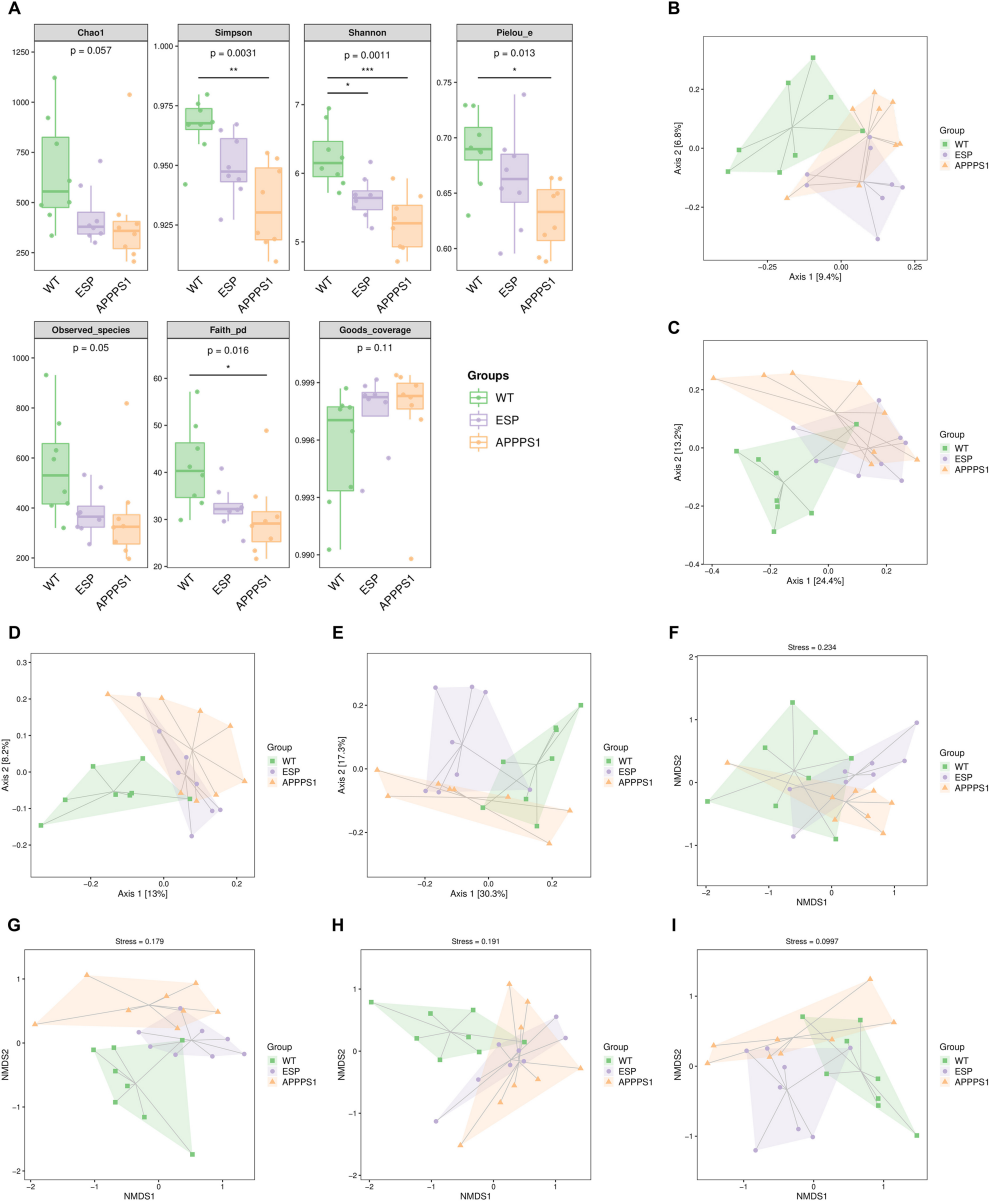
ESP altered fecal microbial diversity of APP/PS1 mice. Fecal microbiota species diversity analyses were conducted by Alpha and Beta diversity analysis. **(A)** The group box plot of the Alpha diversity indices. Each panel corresponds to an Alpha diversity index. In each panel, the horizontal axis represents the grouping label and the vertical axis represents the corresponding Alpha diversity index value. Chao1 and Observed species reflect the richness of the fecal microbial species. Simpson, Shannon, and Faith PD reflect the diversity of the fecal microbial species. Pielou’s E reflects the evenness, while Good’s coverage reflects the cover degree of the fecal microbial species. Kruskal-Wallis test was used for statistical comparison among multiple groups, and *Post hoc* verification of Dunn’s test corrected by Bonferroni was used for comparison between two groups. *p < 0.05, **p < 0.01, and ***p < 0.001. **(B–E)** Scatter plots of principal coordinates analysis (PCoA). Scatter plots of fecal microbiota samples analyzed by Jaccard based PCoA (B), Bray-Curtis based PCoA **(C)**, unweighted UniFrac based PCoA **(D)**, and weighted UniFrac based PCoA **(E)**. Each point in the figure represents a sample, and points of different colors indicate different groups. The percentage in parentheses on the coordinate axis represents the proportion of sample difference data by the corresponding coordinate axis. **(F–I)** Scatter plots of Non-metric Multidimensional Scaling (NMDS). Scatter plots of Jaccard based NMDS **(F)**, Bray-Curtis based NMDS **(G)**, unweighted UniFrac based NMDS **(H)**, and weighted UniFrac based NMDS **(I)**. Each point in the figure represents a sample, and points of different colors indicate different experimental groups. The closer the distance between two points, the smaller the difference in microbial communities between the two groups. *n* = 8.

Fecal microbial community diversity was assessed using Alpha and Beta diversity analyses. Alpha diversity of the fecal microbiota was assessed using Chao1, Simpson, Shannon, Pielou’s E, Observed species, Faith’s PD, and Good’s coverage. The APP/PS1 group showed significantly lower microbial diversity (Simpson, *p* < 0.01; Shannon, *p* < 0.0001; Faith’s PD, *p* < 0.05) and evenness (Pielou’s E, *p* < 0.05) compared to the WT group (Figure 5A). However, no significant differences were observed between the ESP and APP/PS1 groups in any Alpha diversity indices, indicating that ESP intervention did not substantially restore overall microbial diversity in APP/PS1 mice.

Beta diversity was evaluated using Principal Coordinates Analysis (PCoA) and Non-metric Multidimensional Scaling (NMDS) based on Jaccard distance, Bray-Curtis distance, unweighted UniFrac distance, and weighted UniFrac distance. Both analyses revealed distinct clustering among the WT, APP/PS1, and ESP groups, suggesting significant differences in microbial community composition and species abundance (Figures 5B–I). Notably, while ESP did not alter Alpha diversity, it shifted the microbial community structure toward a configuration distinct from both WT and APP/PS1 groups.

**FIGURE 6:**
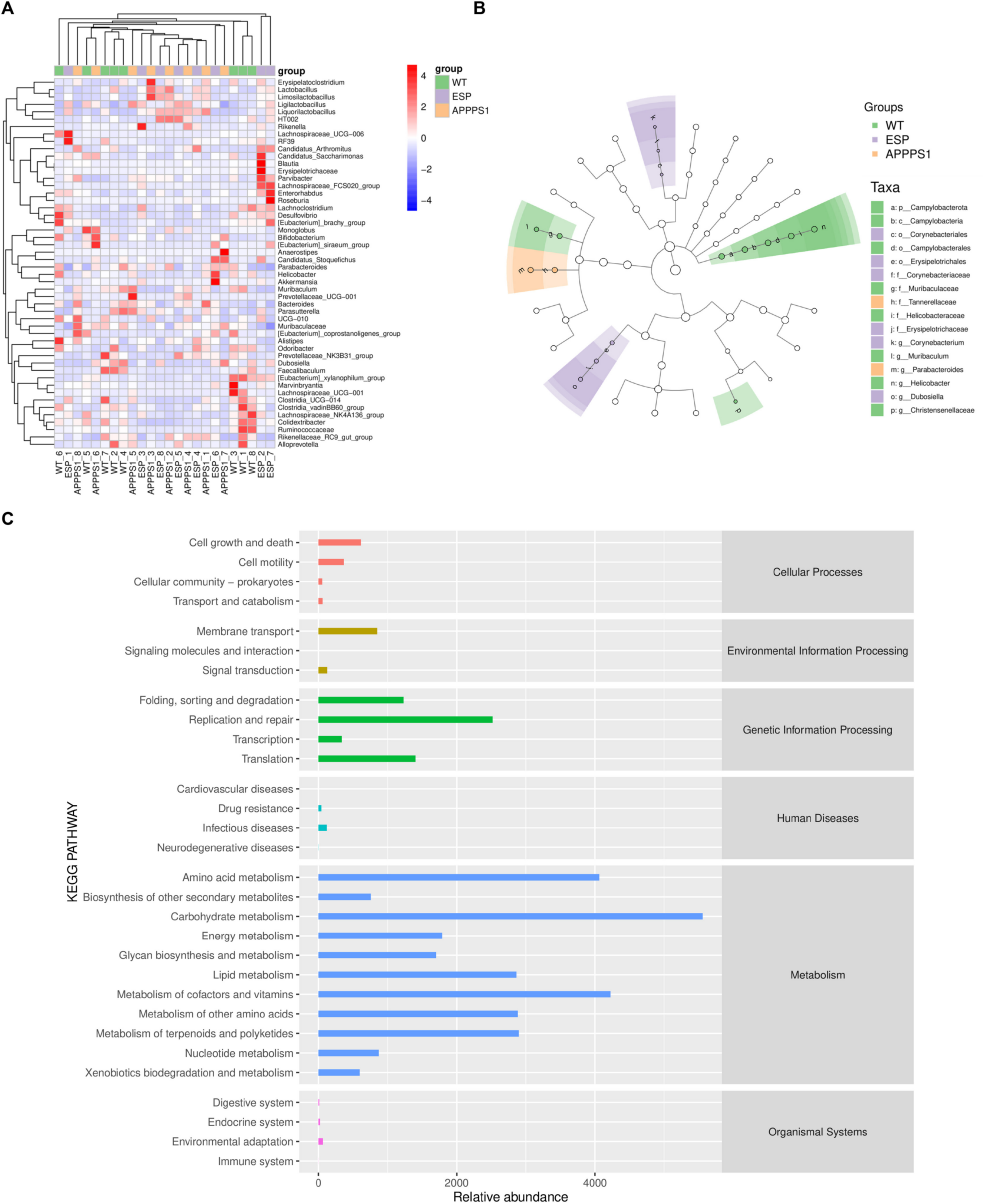
ESP altered specific species of fecal microbiota in APP/PS1 mice. Species difference analysis was conducted among the WT, APP/PS1, and ESP groups. **(A)** Heatmaps of species composition at the genus level of each sample. The samples are clustered according to the euclidean distance of species composition data using Unweighted Pair Group Method with Arithmetic Mean (UPGMA) cluster analysis. Species are clustered using the Pearson correlation coefficient matrix based on their composition data for UPGMA clustering. The samples and species composition were arranged according to the clustering results. Color represents the abundance of fecal microbiota at genus level, red color represents a higher abundance of the genus, while blue color represents a lower abundance of the genus. **(B)** Classification cladogram of intergroup differences based on classification level tree. The cladogram shows the hierarchical relationship of the main taxonomic units from phylum to genus (from inner circle to outer circle). The node size corresponds to the average relative abundance of the classification unit. Green, purple, and orange red represent significant inter group differences. The letters indicate the names of classification units that have significant differences between groups. **(C)** Abundance map of predicted KEGG secondary functional pathways. The horizontal axis represents the relative abundance of functional pathways, while the left vertical axis represents KEGG secondary functional pathways, and the right vertical axis represents the primary pathway to which the secondary pathway belongs. Pathways are categorized into cellular processes, Environmental Information Processing, Genetic Information Processing, Human Diseases, Metabolism, and Organismal Systems. *n* = 8.

To further compare the overall microbial structure and identify key taxonomic shifts, a heatmap was generated based on the relative abundance of the top 50 genera (Figure 6A). The APP/PS1 group exhibited a distinct clustering pattern, characterized by a significant increase in the abundance of harmful genera such as *Bacteroides* and *Parabacteroides*, and a decrease in beneficial genera like *Dubosiella* and *Lachnoclostridium* compared to the WT groups. Notably, ESP treatment reversed the situation.

LEfSe analysis was performed to pinpoint group-specific microbial biomarkers (LDA score > 2.0, *p* < 0.05). At the genus level, *Parabacteroides* was identified as a key biomarker for the APP/PS1 group. In contrast, *Dubosiella* and *Corynebacterium* were significantly enriched in the ESP group (Figure 6B), suggesting that ESP intervention modulated the gut microbiota by suppressing AD-associated taxa and promoting potentially beneficial genera.

Furthermore, to infer the functional consequences of these taxonomic changes, we conducted KEGG pathway analysis based on the metagenomic predictions. Compared to the APP/PS1 group, ESP treatment significantly enriched pathways involved in carbohydrate metabolism and lipid metabolism, which are crucial for host energy homeostasis and neuroprotection (Figure 6C). These functional shifts were potentially linked to the observed increase in *Dubosiella* and *Lachnoclostridium* (Di Martino et al., 2023; Jiang et al., 2025).

### 3.3 ESP modulated the serum metabolites of APP/PS1 mice

**FIGURE 7:**
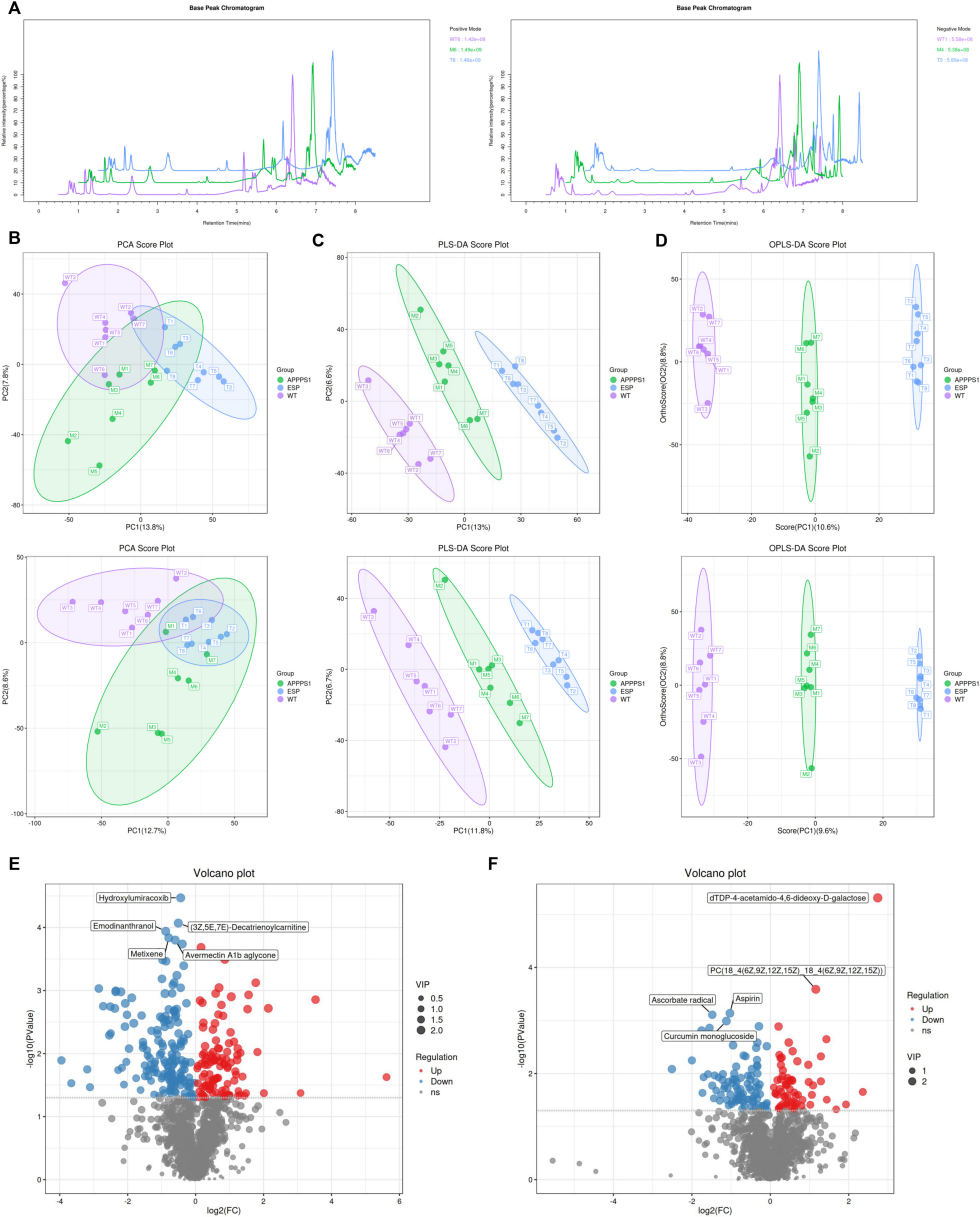
ESP affected metabolic function in APP/PS1 mice. **(A)** Base peak chromatograms (BPC) in positive and negative ion mode. The horizontal axis represents retention time, and the vertical axis represents ion intensity. The upper right corner of the diagram represents the maximum ion intensity of each sample. Different colors represent different groups. **(B)** Principal Component Analysis (PCA) score plots in positive and negative ion mode. The horizontal axis PC1 represents the score value of the first principal component, and the vertical axis PC2 represents the score value of the second principal component. Points represent samples, circles represent 95% confidence intervals, and different colors represent different groups. **(C)** Partial Least Squares Discrimination Analysis (PLS-DA) score plots in positive and negative ion mode. **(D)** Orthogonal Projections to Latent Structures Discriminant Analysis (OPLS-DA) score plots in positive and negative ion mode. The horizontal axis PC1 represents the score value of the first principal component, and the vertical axis OC2 represents the score value of the first orthogonal component. Points represent samples, and different colors represent different groups. The horizontal axis shows inter group differences, and the vertical axis shows intra group differences. The more clustered the intra group samples are, the more dispersed the inter group samples are, indicating that the results are more reliable. **(E,F)** Volcano plots of differential metabolites about ESP group vs. APP/PS1 group **(E)**, and APP/PS1 group vs. WT group **(F)**. The horizontal axis represents the logarithmic value of Log_2_, and the vertical axis represents the logarithmic value of - log10 for *p*-value. Each point represents a metabolite. The larger the absolute value of the horizontal axis, the greater the difference in expression levels of metabolites between the two samples. The larger the vertical axis value, the more significant the differential expression, and the more reliable the differentially expressed metabolites screened. The size of the dots represents the VIP value. The red dots represent differentially upregulated metabolites, the blue dots represent differentially downregulated metabolites, and the gray dots represent no differential metabolites. The figure only displays the *m*/*z-*values of the top five metabolites with the smallest *p*-value. *n* = 7–8.

Untargeted metabolomics was performed to investigate the impact of gut microbiota and their metabolites on host metabolic homeostasis. Representative base peak chromatograms (BPC) of serum samples are displayed in Figure 7A. To assess the systematic metabolic disparities among the WT, APP/PS1, and ESP groups, multivariate statistical analyses were employed. Principal Component Analysis (PCA) revealed a distinct separation between the APP/PS1 and WT groups, indicating a distinct metabolic perturbation in the APP/PS1 model mice. This separation was significantly attenuated in the ESP group, suggesting a partial restoration of metabolic profile (Figure 7B).

To maximize the identification of metabolites that discriminate among groups, supervised models including Partial Least Squares Discrimination Analysis (PLS-DA) and Orthogonal Projections to Latent Structures Discriminant Analysis (OPLS-DA) were constructed. Both PLS-DA and OPLS-DA demonstrated robust model quality (e.g., PLS-DA model: R2Y = 0.999, Q2 = 0.839), and clear segregation of all three groups (Figures 7C,D), confirming significant inter-group metabolic differences.

Subsequently, volcano plots were generated to visualize and identify differential metabolites between specific comparisons, using stringent criteria (|Log_2_FC| > 1.0, *p* < 0.05, VIP > 1). Compared with the APP/PS1 group, the ESP group exhibited significant alterations in 284 metabolites, comprising 114 upregulated and 170 downregulated metabolites (Figures 7E,F). This substantial metabolic shift implies a profound regulatory role of ESP in rectifying the perturbed metabolism associated with the APP/PS1 phenotype.

**FIGURE 8:**
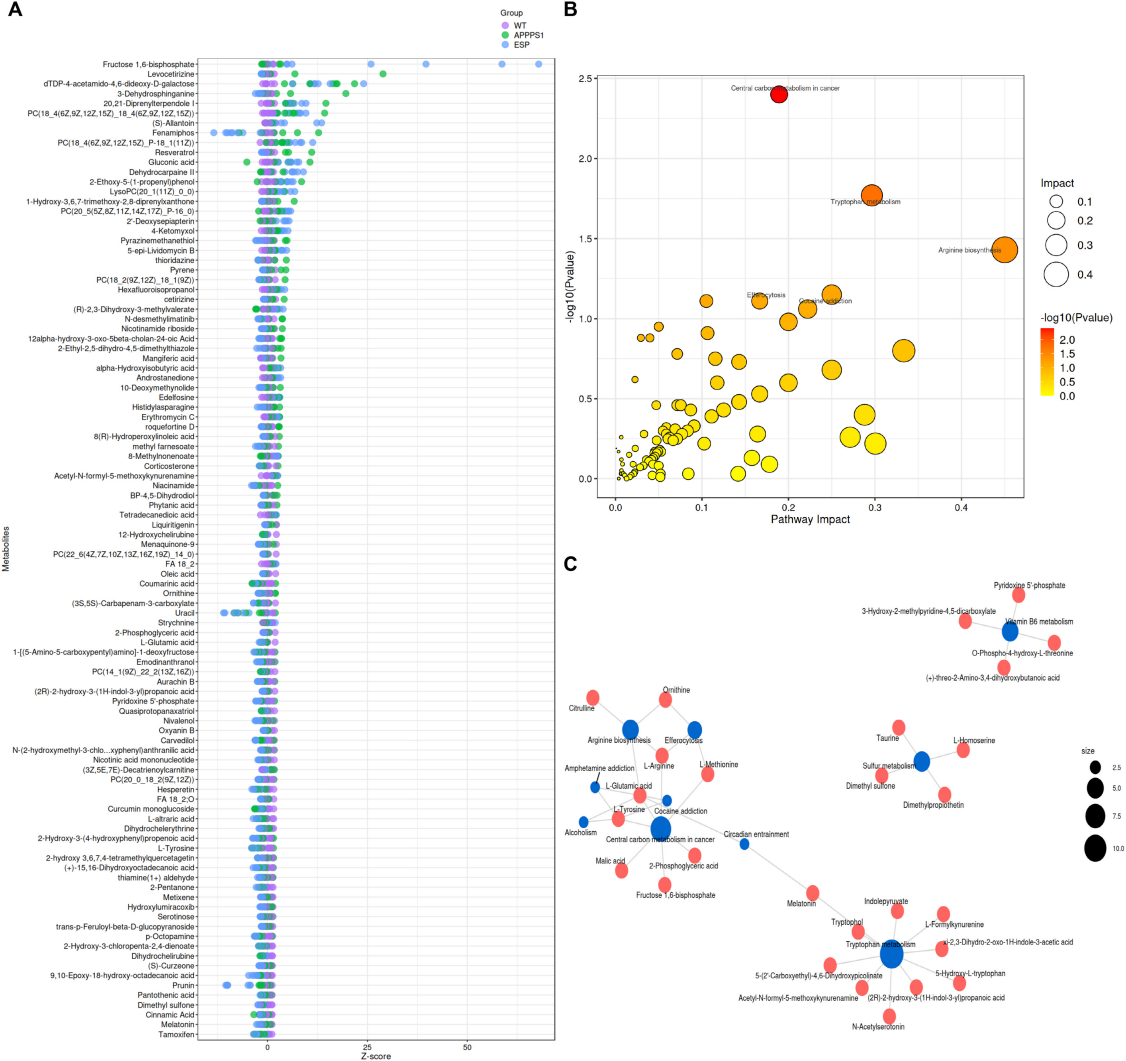
ESP affected differential metabolites in APP/PS1 mice. **(A)** Z-score plot of top 100 differential metabolites. The horizontal axis represents the Z-score value of the relative content of metabolites in the sample after conversion. The vertical axis represents the metabolites name. Different colors represent different groups. The closer the point is to the right, the higher the relative content of metabolites indicated. *n* = 7–8. **(B)** KEGG pathways enrichment bubble plot. The horizontal axis represents the Impact value of enriched metabolic pathways, while the vertical axis represents enriched metabolic pathways. The size of the dot represents the number of metabolites on the pathway. The redder the color, the smaller the *p*-value. **(C)** Network diagram of associated metabolites in top 10 KEGG pathways. The blue dots represent the metabolic pathways, and the red dots represent differential metabolites. The size of the blue dots indicates the number of metabolites connected to the metabolic pathway.

The relative abundance of metabolites across the three groups was compared using Z-score normalization (Sreekumar et al., 2009). Unsupervised hierarchical clustering of these Z-scores revealed a distinct metabolic signature that clearly separated the APP/PS1 group from the WT and ESP groups (Figure 8A), indicating widespread metabolic disruptions in the AD model, and the restoration effect of ESP in metabolites. Among the most significantly altered metabolites, the level of Fructose 1,6-bisphosphate (FBP), a key glycolytic intermediate, was decreased in the APP/PS1 group compared to WT group, and markedly elevated after ESP treatment (*p* < 0.01). The decline in FBP suggests a potential impairment in glycolytic flux, a phenomenon associated with brain energy deficit in AD (Rojas-Pirela et al., 2025).

KEGG pathway enrichment analysis on the differentially abundant metabolites was performed to understand the functional implications. The top 5 significantly enriched pathways were Central carbon metabolism in cancer, Tryptophan metabolism, Arginine biosynthesis, Cocaine addiction, and Efferocytosis (Figure 8B). We focused our interpretation on the first two pathways, given their established links to bioenergetics and neuroprotection in AD.

To visualize the complex interactions between metabolites and pathways, an integrated network was constructed (Figure 8C). This network pinpointed Central carbon metabolism in cancer pathway (involving FBP, L-Tyrosine, etc.) and the Tryptophan metabolism pathway (involving Melatonin, and multiple kynurenine/indole derivatives) as interconnected hubs of disturbance. The coordinated dysregulation of metabolites within these hubs suggests a coupled disruption in cellular energy production (via central carbon metabolism) and neurotransmitter synthesis (via tryptophan metabolism) in APP/PS1 mice (Lian et al., 2025; Williams et al., 2020).

**FIGURE 9:**
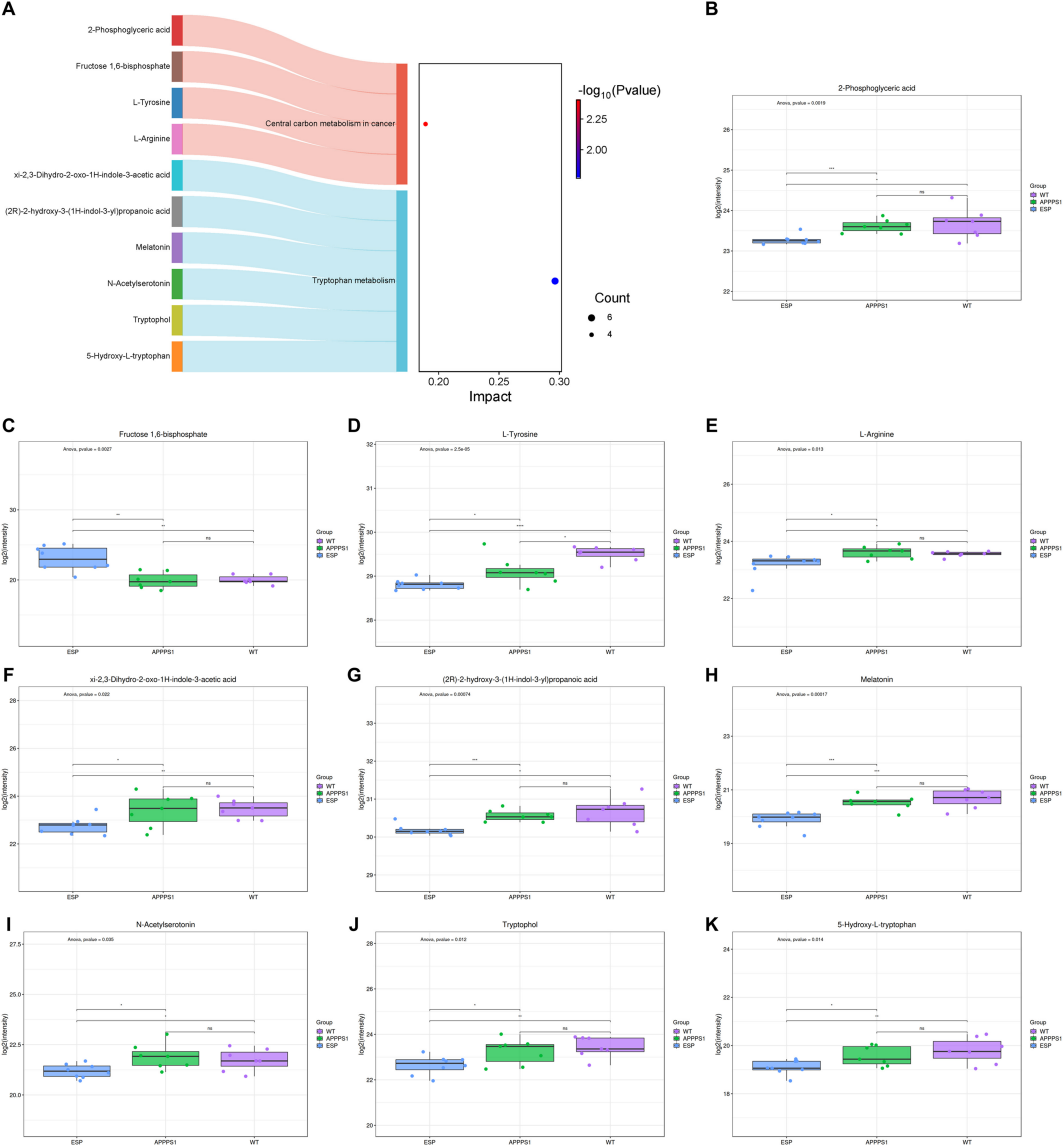
ESP regulated the specific metabolites of the top two metabolic pathways in APP/PS1 mice. **(A)** Sankey bubble chart of the top 2 metabolic pathways. The Sankey diagram on the left represents the metabolites contained in each metabolic pathway. In the bubble chart on the right, the size of the bubble indicates the number of metabolites belonging to the metabolic pathway, and the redder the color, the smaller the *p*-value. **(B–K)** Box plots of representative metabolites in the top two metabolic pathways, including 2-PG **(B)**, FBP **(C)**, L-Tyrosine **(D)**, L-Arginine **(E)**, OAA **(F)**, ILA **(G)**, Melatonin **(H)**, NAS **(I)**, Tryptophol **(J)**, and 5-HTP **(K)**. The horizontal axis represents different groups, while the vertical axis represents the range of metabolites quantitative values. *p < 0.05, **p < 0.01, ***p < 0.001, and ****p < 0.0001. *n* = 7–8.

To further investigate the impact of ESP treatment on metabolic profiles in APP/PS1 mice, a Sankey diagram analysis was performed to visualize the associations between differentially expressed metabolites and two key pathways: Central carbon metabolism in cancer and Tryptophan metabolism (Figure 9A). Subsequent box plots revealed the relative abundance of individual metabolites across the three groups (Figures 9B–K). The results demonstrated that ESP treatment significantly modulated multiple metabolites involved in both pathways. In Central carbon metabolism in cancer, 2-Phosphoglyceric acid (2-PG), L-Tyrosine, and L-Arginine were reduced while FBP was elevated. In Tryptophan metabolism, levels of xi-2,3-Dihydro-2-oxo-1H-indole-3-acetic acid (OAA), (2R)-2-hydroxy-3-(1H-indol-3-yl)propanoic acid (ILA), Melatonin, N-Acetylserotonin (NAS), Tryptophol, and 5-Hydroxy-L-tryptophan (5-HTP), were decreased. These metabolic changes will be further discussed in the context of ESP’s potential mechanisms against AD pathology.

### 3.4 Correlation analysis of metabolites and gut microbiota

**FIGURE 10:**
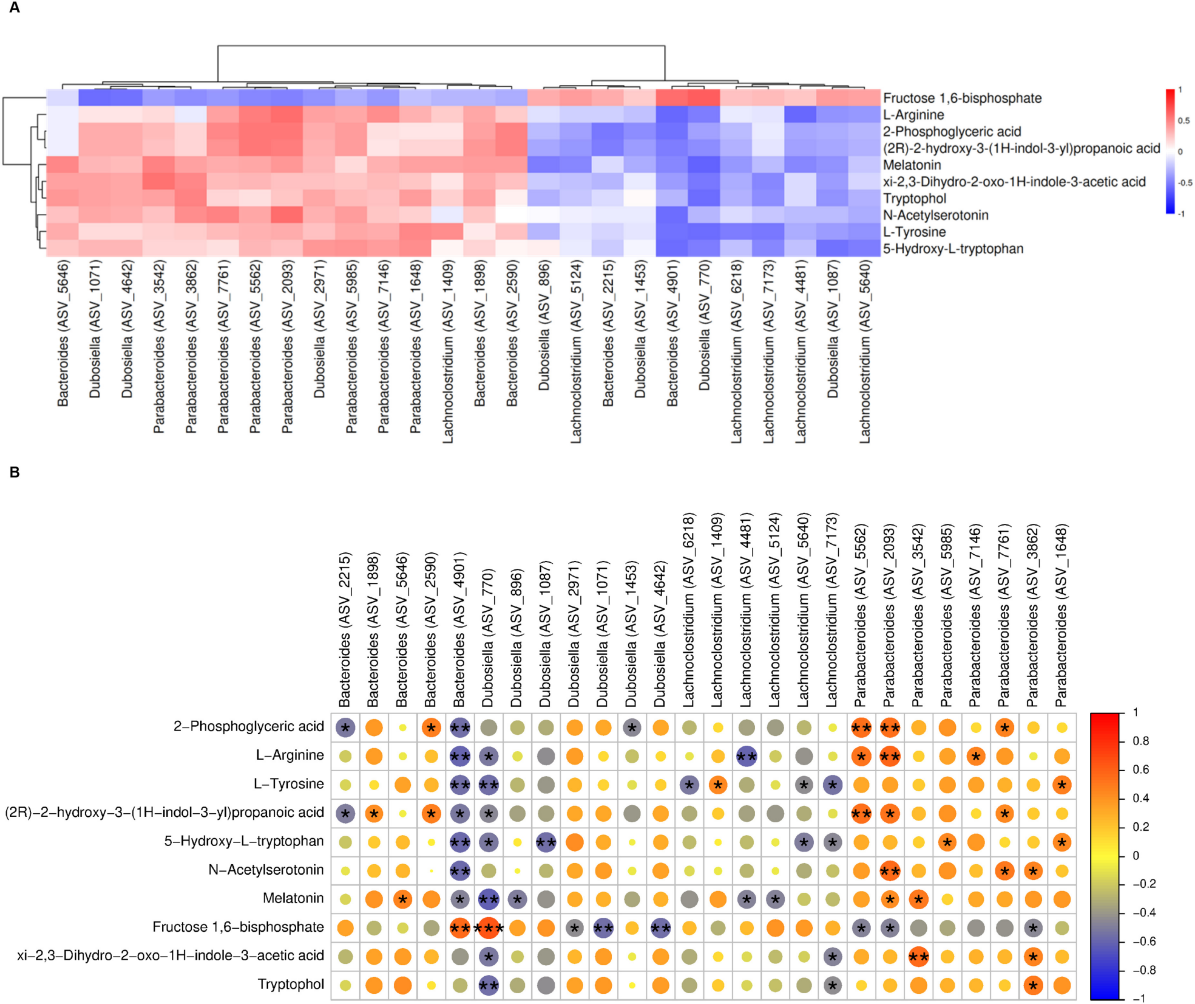
Correlation analysis between specific metabolites and microbiota. Correlation heatmap **(A)** and correlation coefficient plot **(B)** between specific metabolites and microbiota. The X-axis represents microbiota, while the Y-axis represents metabolites. The color intensity of rectangle and circle, and the size of the circle indicate the magnitude of the correlation coefficient between metabolites and microbiota. *p < 0.05, **p < 0.01, and ***p < 0.001.

Spearman correlation analysis revealed significant associations between specific serum metabolites and ASVs from several bacterial genera, including *Bacteroides*, *Dubosiella*, *Lachnoclostridium*, and *Parabacteroides* (Figures 10A,B). Focusing on FBP, we observed distinct correlations with ASVs within these genera. Specifically, *Bacteroides* ASV_4901 was positively correlated with FBP (ρ = + 0.577, *p* < 0.01). Within the genus *Dubosiella*, ASV_770 exhibited a strong positive correlation with FBP (ρ = + 0.656, *p* < 0.001), whereas ASV_1071, ASV_4642, and ASV_2971 showed significant negative correlations (ρ = −0.557 to −0.428, all *p* < 0.05). Among *Parabacteroides* ASVs, ASV_5562, ASV_2093, and ASV_3862 showed negative correlations with FBP (ρ range: −0.477 to −0.453, all *p* < 0.05). Notably, the strongest association identified was the positive correlation between *Dubosiella* ASV_770 and FBP, suggesting a potentially key microbial player in FBP metabolism or response.

## 4 Discussion

The mechanisms underlying the potential neuroprotective effects of ESP, a traditional Tibetan medicine, against AD remain unclear. Herein, an integrated multi-omics approach, combining 16S rRNA gene sequencing and untargeted metabolomics, were employed to investigate the mechanisms underlying ESP therapeutic effects on AD. Our study demonstrated that ESP administration significantly ameliorated cognitive deficits and key AD-like neuropathology in the APP/PS1 mice. More importantly, our findings showed that these neuroprotective effects were accompanied by a comprehensive remodeling of the gut microbiota, consequential shifts in host metabolism, and a marked reduction in neuroinflammation. These multi-omics findings reveal that ESP exerts anti-AD benefits by modulating the gut microbiota-metabolite-brain axis.

We first demonstrated that ESP treatment effectively restored gut microbial homeostasis in APP/PS1 mice. Specifically, ESP reduced the relative abundance of *Bacteroides* and *Parabacteroides*, and increased the abundance of *Dubosiella* and *Lachnoclostridium*. The interpretation of *Bacteroides* alterations in AD has been contentious. While several studies report a positive correlation between *Bacteroides* abundance and AD (Haran et al., 2019; Saji et al., 2019; Vogt et al., 2017), other reports show decreased levels in some patient cohorts (Murray et al., 2022). This variability may stem from differences in disease stage, heterogeneity of patient populations (age, diet, medication), or specific effects of species/strains below the genus level. In our model, the observed decrease in *Bacteroides* following ESP treatment correlated with cognitive improvement. In contrast, the role of *Parabacteroides* as a potentially detrimental genus is more consistently supported; its abundance has been positively associated with neuroinflammation and cognitive impairment (Chevalier et al., 2025; Khine et al., 2020; Noble et al., 2021). Conversely, ESP increased *Dubosiella* and *Lachnoclostridium*, which are positively correlated with cognitive function and immune homeostasis (Chen et al., 2024; Di Martino et al., 2023; Jiang et al., 2025; Wu et al., 2024; Yuan et al., 2025; Zhu et al., 2025). Collectively, these microbial shifts suggest that ESP drives the gut ecosystem toward an anti-inflammatory and metabolically favorable profile, providing a microbial basis for the observed neuroprotection. We therefore propose that ESP-induced modulation of the gut microbiota serves as a primary driver for the downstream host metabolic alterations we observed.

To directly link these microbial changes to host metabolic reprograming, we performed untargeted metabolomics, which revealed a profound restructuring of energy metabolism in APP/PS1 mice that was partially normalized by ESP. Given that disrupted energy metabolism is an early hallmark of AD (Du et al., 2021; Liu Z. et al., 2025), we focused on key glycolytic intermediates. The level of FBP, a critical glycolytic intermediate capable of promoting protective macrophage differentiation and suppressing microglia-mediated neuroinflammation (Kim et al., 2012; Zhou et al., 2022), was decreased in APP/PS1 mice compared to WT mice, aligning with impaired brain glucose metabolism in AD (Kabonick et al., 2025; Rojas-Pirela et al., 2025). Notably, ESP intervention partially restored FBP levels, which may contribute to its anti-inflammatory effects. In contrast, the level of 2-PG, a downstream metabolite of FBP, was also decreased in APP/PS1 mice—consistent with previous reports (Bergau et al., 2019; Chen J. et al., 2025)—yet was further reduced upon ESP treatment. This opposing shift between FBP and 2-PG suggests that ESP does not globally suppress glycolytic flux but rather selectively modulates specific enzymatic activities, such as aldolase, downstream of FBP (Kulow et al., 2025). This nuanced regulation implies that ESP reprograms glucose metabolism toward alternative fates, potentially optimizing limited glucose utilization or redirecting flux into neuroprotective pathways like the pentose phosphate pathway (Jimenez-Blasco et al., 2024).

Beyond glycolysis, ESP modulated amino acid metabolic pathways intimately linked to AD pathology. Elevated L-Arginine is associated with Aβ deposition and neuroinflammation (Louw et al., 2007; Ju et al., 2022), and ESP significantly reduced its abnormally elevated levels in APP/PS1 mice, representing a plausible anti-pathological mechanism. ESP also reduced L-Tyrosine levels. Intriguingly, recent evidence indicates that cellular tyrosine concentration can regulate the nuclear translocation and DNA-repair function of tyrosyl-tRNA synthetase (TyrRS) (Jhanji et al., 2022). These data raise the hypothesis that ESP may enhance neuronal DNA repair capacity by promoting TyrRS nuclear translocation through lowering L-Tyrosine, a direction warranting further targeted investigation.

The multi-omics integration is particularly evident in tryptophan metabolism, where microbial and host pathways converge. During the pathogenesis of AD, neuroinflammation activation leads to an imbalance in tryptophan metabolism (Li et al., 2022). Our metabolomics analysis revealed comprehensive reprograming of this pathway by ESP, with significant alterations in levels of NAS, melatonin, 5-HTP, and several microbiota-derived indole derivatives including tryptophol, ILA, and OAA. Importantly, ESP reduced both NAS and melatonin levels in APP/PS1 mice. Given that ESP effectively inhibited Aβ accumulation, P-tau pathology, and neuroinflammation, we interpret the decline in these neuroprotective molecules not as an adverse effect, but rather as a homeostatic recalibration: attenuated pathological stimuli diminish the need for compensatory upregulation of these endogenous protectants (Bocheva et al., 2024; Hornedo-Ortega et al., 2018; Hossain et al., 2019; Kang et al., 2023; Long and Holtzman, 2019; Mondanelli et al., 2020). The concurrent reduction in 5-HTP further aligns with an overall shift toward a less inflammatory and oxidative metabolic state (Liu C. et al., 2025; Maffei, 2020).

Directly bridging microbial remodeling to metabolic output, ESP significantly decreased levels of the gut microbiota-dependent metabolites ILA, OAA, and tryptophol. This provides direct functional evidence that the ESP-induced correction of gut dysbiosis translates into altered host metabolism (Ephraim et al., 2020; Kim et al., 2024; Savonije and Weaver, 2023). As these indole derivatives modulate systemic immunity and microglial activity (Dos Santos et al., 2022; Wang et al., 2024; Zhang et al., 2023), their altered abundance mechanistically connects gut microbial remodeling to the anti-inflammatory and neuroprotective effects observed.

Our findings align with the established literature linking gut dysbiosis to AD pathogenesis and support microbiota-targeted interventions. Studies using fecal microbiota transplantation in AD rodent models and patients have demonstrated that altering the gut microbiota can influence disease progression (Hazan, 2020; Liu X. et al., 2025; Park et al., 2021; Shen et al., 2020). However, the specific role of traditional medicine formulations like ESP within gut-brain axis remains largely unexplored. Our study systematically elucidates, through multi-omics integration, how ESP ameliorates cognitive deficits and neuropathology by concurrently restoring gut microbial homeostasis and modulating host metabolite profiles.

In summary, ESP orchestrates a multi-faceted response against AD pathology. Strong neuroinflammation, triggered by accumulation of Aβ and P-tau in AD model mice, can alter the intestinal microenvironment through pathways such as cytokine release, thereby affecting the host metabolic environment (Courade et al., 2025; Popescu et al., 2024). By mitigating central proteotoxicity and neuroinflammation, ESP reduces the demand for compensatory metabolic neuroprotection. In parallel, ESP likely restores systemic and intestinal homeostasis, in part through modulating the gut microbiota and metabolic output. This dual action—centrally and peripherally—converges to break the vicious cycle of neuroinflammation, microbiota dysbiosis and metabolic dysregulation, exemplifying a systemic therapeutic approach aligned with the holistic principles of traditional medicine.

However, this study has several limitations that should be addressed in future research. First, our findings are primarily derived from a single transgenic AD mouse model, which may not fully represent the AD pathogenesis (e.g., tauopathy models). Consequently, the generalizability of ESP’s efficacy across other AD models remains to be established. Second, although ESP demonstrates neuroprotective properties, the specific bioactive compounds responsible for these effects have not yet been isolated or functionally validated. Third, while our data suggest a correlation between gut microbiota modulation and cognitive improvement, definitive causal evidence is still lacking. Future studies should employ FMT and utilize germ-free or antibiotic-treated animal models to definitively establish the indispensability of the gut microbiota in ESP-mediated neuroprotection. Fourth, the precise mechanisms linking ESP-induced microbial changes to neuroinflammation and cognitive function are unclear. Integrating multi-omics approaches (e.g., brain metabolomics with transcriptomics/proteomics) would be employed to elucidate the downstream signaling pathways involved. Ultimately, to translate these preclinical findings into clinical applications, well-controlled clinical trials in patients are necessary.

## Data Availability

The original contributions presented in the study are included in the article/Supplementary material, further inquiries can be directed to the corresponding author.

## Footnotes

1
http://www.hmdb.ca

2
http://www.massbank.jp/

3
http://www.lipidmaps.org

4
https://www.mzcloud.org

5
https://www.genome.jp/kegg/

6
https://www.biodeep.cn

7
https://www.bioinformatics.com.cn

## Supplementary material

Supplementary Table 1 Raw data of fecal microbiota.

Supplementary Table 2 Raw data of serum metabolites.
